# Supplementary material for: Ricin Antibodies’ Neutralizing Capacity against Different Ricin Isoforms and Cultivars
Source: Toxins (Basel). 2021 Jan 29;13(2):100. doi: 10.3390/toxins13020100 (PMC7911099; doi:10.3390/toxins13020100)
Supplement: Supplementary file 1 [file toxins-13-00100-s001.pdf]

Supplementary Materials: Ricin Antibodies' Neutralizing Capacity Against Different Ricin Isoforms and Cultivars

Maria Lucia Orsini Delgado, Arnaud Avril, Julie Prigent, Julie Dano, Audrey Rouaix, Sylvia Worbs, Brigitte G. Dorner, Clémence Rougeaux, François Becher, François Fenaille, Sandrine Livet, Hervé Volland, Jean-Nicolas Tournier and Stéphanie Simon

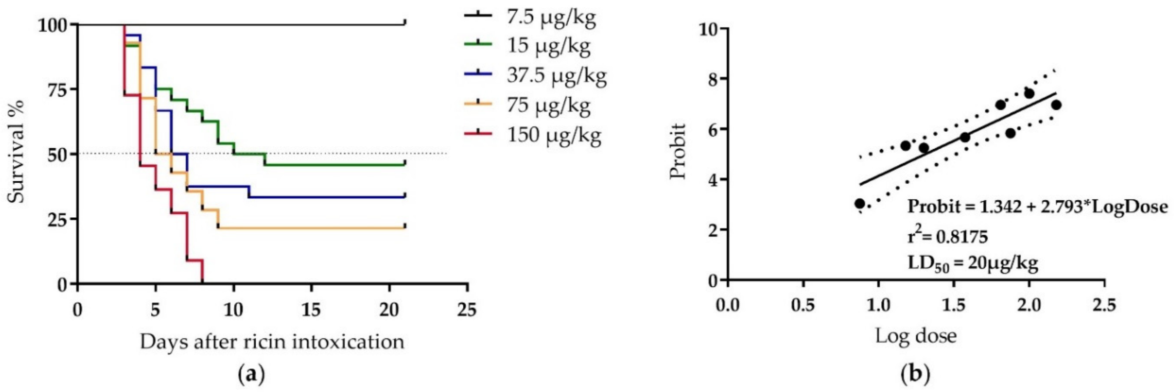

Figure S1. In vivo 50% lethal dose (LD_{50}) of the equimolar solution of ricin D/E. Anesthetized female BALB/cJ mice were poisoned by the intranasal route with different doses of an equimolar solution of ricins D and E. Clinical signs and survival were recorded for 21 days, and when needed an ethical end point was applied. (**a**) Survival curves, percentage survival for mice receiving 7.5 µg/kg (black solid line), 15 µg/kg (green solid line), 37.5 µg/kg (blue solid line), 75 µg/kg (orange solid line), or 150 µg/kg (red solid line). (**b**) Determination of LD_{50} using the Miller and Tainter method as described by Randhawa 2009 [23], for which a probit value of 5 corresponds to 50% survival.

Table S1. Determination of residual *R. communis* agglutinin (RCA120), ricins D and E in the different ricin purified fractions.

	Residual RCA120/Ricin E *	Residual RCA120 **	Residual Ricin D ***
Ricin D	<1%	<1%	100%
Ricin E1	113 ± 15%	12 ± 5%	<1%
Ricin E2	87 ± 14%	9 ± 9%	<1%

* Signal of RCA120/ricin E peptides in "ricin E1" and "ricin E2" was used as a reference (mean of E1 and E2); ** signal of RCA120 peptides in a standard of RCA120 was used as a reference; *** signal of ricin D peptide in "ricin D" was used as a reference. Mean values of two experiments.
